# Early AbobotulinumtoxinA (Dysport^®^) in Post-Stroke Adult Upper Limb Spasticity: ONTIME Pilot Study

**DOI:** 10.3390/toxins10070253

**Published:** 2018-06-21

**Authors:** Raymond L Rosales, Jovita Balcaitiene, Hugues Berard, Pascal Maisonobe, Khean Jin Goh, Witsanu Kumthornthip, Mazlina Mazlan, Lydia Abdul Latif, Mary Mildred D. Delos Santos, Chayaporn Chotiyarnwong, Phakamas Tanvijit, Odessa Nuez, Keng He Kong

**Affiliations:** 1 Centre for Neurodiagnostic and Therapeutic Services (CNS), Metropolitan Medical Centre, Manila 1012, Philippines; marymildred8888@yahoo.com; 2 Department of Neurology & Psychiatry, Faculty of Medicine and Surgery, University of Santo Tomas, Manila 1008, Philippines; 3 Ipsen Group, Boulogne-Billancourt, 92100 Paris, France; jovita.balcaitiene@gmail.com (J.B.); hugues.berard@ipsen.com (H.B.); pascal.maisonobe@ipsen.com (P.M.); 4 Division of Neurology, Department of Medicine, University of Malaya Medical Centre, Kuala Lumpur 59100, Malaysia; gohkj@ummc.edu.my; 5 Department of Rehabilitation Medicine, Siriraj Hospital, Mahidol University, Bangkok 10700, Thailand; wkumthornthip@yahoo.com (W.K.); hedhom2000@hotmail.com (C.C.); siphakamas@gmail.com (P.T.); 6 Department of Rehabilitation Medicine, Faculty of Medicine, University of Malaya, Kuala Lumpur 50603, Malaysia; mazlinamazlan@ummc.edu.my (M.M.); lydia@ummc.edu.my (L.A.L.); 7 Department of Rehabilitation Medicine, Tan Tock Seng Hospital, Novena 308433, Singapore; nuez_odessa_setiota@ttsh.com.sg (O.N.); keng_he_kong@ttsh.com.sg (K.H.K.)

**Keywords:** abobotulinumtoxinA, botulinum toxin type A, upper limb spasticity, post-stroke, early use, ONTIME

## Abstract

The ONTIME study investigated whether early post-stroke abobotulinumtoxinA injection delays appearance or progression of upper limb spasticity (ULS) symptoms. ONTIME (NCT02321436) was a 28-week, exploratory, double-blind, randomized, placebo-controlled study of abobotulinumtoxinA 500U in patients with ULS (Modified Ashworth Scale [MAS] score ≥ 2) 2–12 weeks post-stroke. Patients were either symptomatic or asymptomatic (only increased MAS) at baseline. Primary efficacy outcome measure: time between injection and visit at which re-injection criteria were met (MAS ≥ 2 and ≥1, sign of symptomatic spasticity: pain, involuntary movements, impaired active or passive function). Forty-two patients were randomized (abobotulinumtoxinA 500U: *n* = 28; placebo: *n* = 14) with median 5.86 weeks since stroke. Median time to reach re-injection criteria was significantly longer for abobotulinumtoxinA (156 days) than placebo (32 days; log-rank: *p =* 0.0176; Wilcoxon: *p =* 0.0480). Eleven (39.3%) patients receiving abobotulinumtoxinA did not require re-injection for ≥28 weeks versus two (14.3%) in placebo group. In this exploratory study, early abobotulinumtoxinA treatment significantly delayed time to reach re-injection criteria compared with placebo in patients with post-stroke ULS. These findings suggest an optimal time for post-stroke spasticity management and help determine the design and sample sizes for larger confirmatory studies.

## 1. Introduction

Spasticity, arising from involuntary activation of muscles, may lead to pain, disability, functional impairment, and eventually, contractures [1]. Post-stroke spasticity (PSS) occurs in one third of stroke survivors [1], and has consistently been demonstrated to negatively impact patients’ quality of life and increase the number of falls and fractures, as well as caregiver burden [2,3,4]. Additionally, direct costs are quadrupled in stroke survivors with spasticity vs. those without [5]. ‘Symptomatic’ patients with upper limb spasticity (ULS) present with symptoms such as impaired passive and active function, pain, and associated reactions [6]. Time to development of symptomatic PSS ranges from 3 to 18 months [6,7,8,9,10]; however, some studies have shown muscle tone changes in the affected limb within 3 weeks post-stroke [11,12,13].

Botulinum toxin A (BoNT-A) safety and efficacy in chronic focal spasticity treatment is well established [14,15,16]. BoNT-A is not usually initiated until muscle overactivity is demonstrated [17], thus, data regarding early PSS treatment are limited. However, as far back as 2010, an international consensus statement advocated the role of early BoNT-A injection in preventing contracture development, with potential to unmask active functional improvement [15]. Early BoNT-A may improve hypertonicity, passive function, and pain in upper and lower limb spasticity [18]; however, the impact of BoNT-A on symptomatic spasticity progression has not been evaluated. The ABCDE-S study investigated efficacy of early (≤12 weeks post-stroke) abobotulinumtoxinA on muscle tone in patients with post-stroke ULS [13]. ONTIME is an exploratory study, and the first to investigate whether early abobotulinumtoxinA not only improves muscle tone, but also delays the time to symptom development or progression in PSS. This study will also provide additional supportive data to determine the design and sample size for further confirmatory studies.

### Objectives

Primary objective: assessment of time between upper limb (UL) injection of abobotulinumtoxinA (≤12 weeks post-stroke) and the appearance or progression of symptomatic ULS. For this, ONTIME used a composite index, consisting of pre-selected, clinic-based, observable re-injection criteria, focused on hypertonicity and functional symptoms of spasticity.

Secondary objectives: assessments of muscle tone in primary targeted muscle group (PTMG), UL active motor function, time to reach re-injection criteria stratified by baseline symptomatic/asymptomatic spasticity and global assessment of change. Concomitant non-drug therapy sessions were recorded.

## 2. Results

### 2.1. Baseline Characteristics and Patient Disposition

**Figure 1:**
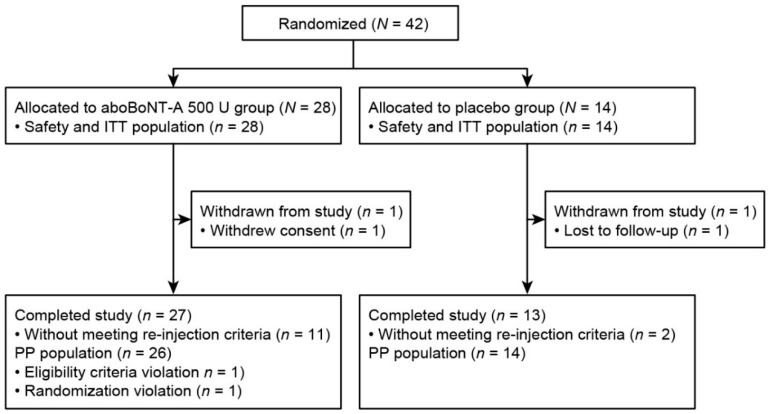
Patient disposition. aboBoNT-A, abobotulinumtoxinA; ITT, intention-to-treat; PP, per-protocol.

Forty-two patients with ULS were recruited and randomized (abobotulinumtoxinA 500U, *n* = 28; placebo, *n* = 14). Safety and intention-to-treat (ITT) populations included all randomized and injected patients (*n* = 42). The per-protocol population included 40 (95.2%) patients. Two (4.8%) patients withdrew from the study: one patient (abobotulinumtoxinA) withdrew consent, and one (placebo) was lost to follow-up and excluded (Figure 1).

Baseline characteristics were well-matched between treatment groups (Table S1). Mean time post-stroke was 6.18 and 6.52 weeks for abobotulinumtoxinA and placebo, respectively. Elbow flexors were the most commonly selected PTMG. Patients mostly presented with baseline symptomatic spasticity (76.2%), i.e., affected by impaired passive function (64.3%; Likert score ≥ 1), active function (57.1%), involuntary movements (47.6%), and/or pain (38.1%; Numeric Pain Rating Scale [NPRS] score ≥ 4), in addition to hypertonicity (MAS ≥ 2; Table S1). Symptom presentation varied between treatment groups and both groups recorded mild pain (mean NPRS score: 3.1) (Table S1).

### 2.2. Time to Re-Injection

Median (95% confidence interval [CI]) time between injection and meeting re-injection criteria was significantly longer for abobotulinumtoxinA (156.0 [86.0–206.0] days) vs. placebo (32.0 [29.0–114.0] days; log-rank: *p =* 0.0176; Wilcoxon: *p =* 0.0480).

**Figure 2:**
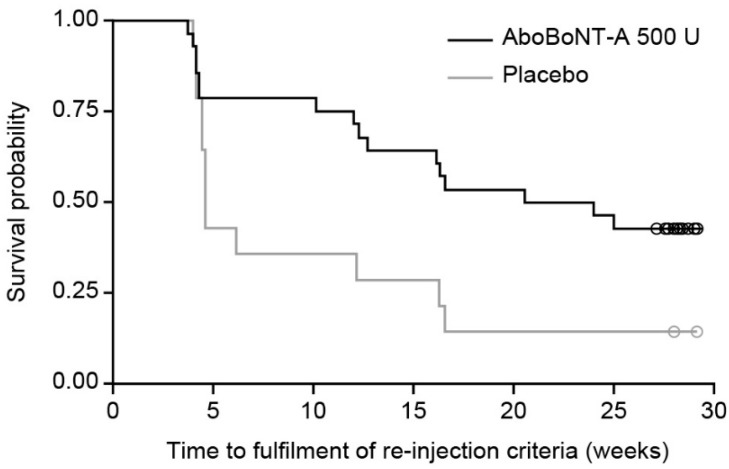
Time to fulfilment of re-injection criteria—KM analysis (ITT population). Circles indicate patients who had not met re-injection criteria at their last study visit. AboBoNT-A, abobotulinumtoxinA; ITT, intention to treat; KM, Kaplan–Meier.

For abobotulinumtoxinA, 20/28 patients (71.4%) did not meet re-injection criteria until after Week 12, vs. 5/14 (35.7%) for placebo (Figure 2). At study end, 11 (39.3%) patients in the abobotulinumtoxinA group had not met re-injection criteria, vs. 2 (14.3%) for placebo.

For the symptomatic spasticity cohort, a significant difference was observed between abobotulinumtoxinA and placebo (130.0 [30.0–206.0] vs. 32.0 [28.0–85.0] days, respectively; log-rank: *p =* 0.0384; Wilcoxon: *p =* 0.0798). The asymptomatic cohort had insufficient patients (*n* = 10) for robust statistical testing.

### 2.3. Assessment of Change in Muscle Tone

**Figure 3:**
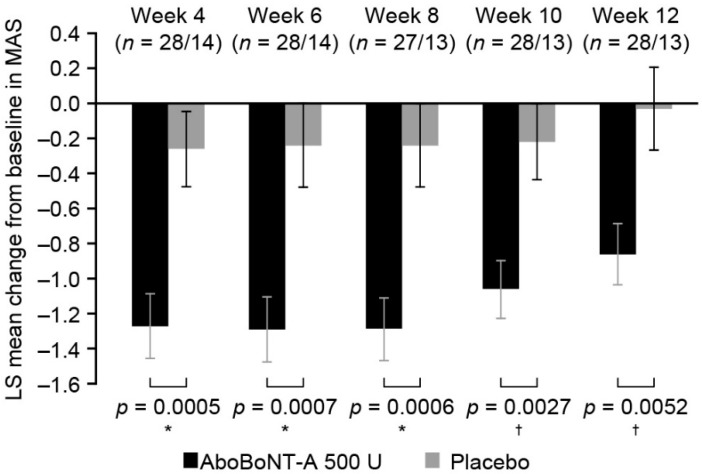
MAS change from baseline to each visit (ITT population). Values are presented as the least squares mean changes from baseline (standard error) in MAS score. * *p* ≤ 0.01 and † *p* ≤ 0.001, *p*-values are based on an analysis of variance performed at each visit. AboBoNT-A, abobotulinumtoxinA; LS, least squares; MAS, Modified Ashworth Scale; *n*, number of patients in aboBoNT-A group/placebo group at each visit.

Differences in muscle tone between abobotulinumtoxinA and placebo were statistically significant at first post-baseline assessment (Week 4) to Week 12, with the maximal decrease in muscle tone observed at Weeks 6 and 8 with abobotulinumtoxinA (Figure 3). At Week 12, difference in MAS score was −0.83 (−1.39, −0.26; *p =* 0.0052). After Week 12, no robust calculation could be completed due to low placebo group patient numbers.

### 2.4. Assessment of Motor Function Recovery

No statistically significant differences in motor recovery scores were observed. As most placebo group patients met re-injection criteria at Week 4 (Figure 2), motor function was not assessed at subsequent visits. At Week 4, change from baseline was numerically higher for abobotulinumtoxinA vs. placebo (6.4 [2.5] vs. 5.6 [4.4], *p =* 0.8754) and motor function improved with abobotulinumtoxinA until Week 12 (11.1 [3.5]).

### 2.5. Changes in Global Assessment

No statistically significant association between treatment and global assessment of changes at last visit was identified (*p =* 0.6128); however, numerically higher proportions of patients treated with abobotulinumtoxinA were assessed as ‘better’ or ‘much better’ (Table 1). Placebo group data were limited, due to meeting re-injection criteria.

### 2.6. Concomitant Therapy

Thirty-nine (92.9%; 89.3% for abobotulinumtoxinA vs. 100% for placebo) patients received concomitant non-drug therapies for PSS in their affected UL, including physiotherapy (85.7%) and occupational therapy (31.0%). Mean duration of physiotherapy was 157.9 days with abobotulinumtoxinA and 126.1 days with placebo (Table S2). Patients receiving abobotulinumtoxinA had a longer duration of therapy vs. placebo, as most placebo group patients met re-injection criteria before Week 12.

Concomitant medications received during the study are described in Table S3.

### 2.7. Safety Assessment

Twenty-three treatment-emergent adverse events (TEAEs) occurred in 12 patients (Table 2); most were mild-to-moderate intensity. One abobotulinumtoxinA group patient reported two severe TEAEs (pain and hypertensive crisis), neither were considered related to treatment by the investigator. Three abobotulinumtoxinA group patients reported four serious adverse events (AEs) during the study. Two patients reported head injury following a fall, one reported asthma exacerbation, and one reported pneumonia. All patients recovered and none were considered related to study treatment. No AEs led to premature withdrawal and no deaths were reported. All variations in vital signs were considered within normal range.

## 3. Discussion

The ONTIME exploratory study assessed whether abobotulinumtoxinA 500U, administered 2–12 weeks after stroke, delays development of symptomatic spasticity in adult patients with increased muscle tone (MAS ≥ 2). Baseline MAS ≥ 2 was based on results from a previous study [11]. AbobotulinumtoxinA significantly prolonged time to fulfilment of re-injection criteria compared with placebo, with abobotulinumtoxinA-treated patients having an additional 124 days before they met re-injection criteria. At Week 28, 39.3% of patients (*n* = 11) who received abobotulinumtoxinA had not reached re-injection criteria, vs. 14.3% (*n* = 2) for placebo. These substantial benefits of abobotulinumtoxinA over placebo were observed despite any potential influence of the high level of concomitant non-drug therapy use in each group. AEs were similar between groups and there were no related TEAEs.

While based on goals, a physician’s decision to inject BoNT-A is not driven by increased muscle tone alone but by signs of impaired function, pain, or both, which can differ in presentation among patients. Thus, the novel composite re-injection criteria used in ONTIME, combining hypertonicity and associated symptoms of spasticity, may make the present results particularly relevant to clinical practice. Spasticity-related clinical accompaniments of the composite re-injection criteria were included, based on previous findings that identified pain, involuntary movements, and impaired active and passive function as key treatment goal areas for patients with post-stroke ULS [19]. In this present study, 76% of patients were symptomatic at baseline (2–12 weeks post-stroke), with 38–64% exhibiting ≥1 item of symptomatic spasticity, in addition to hypertonicity. The prevalence and heterogenic presentation of symptoms observed highlights the need for composite, patient-centered outcome measurements and the importance of early intervention.

The long time to reach re-injection criteria observed during the ONTIME study (156.0 days for abobotulinumtoxinA vs. 32.0 days for placebo) is supported by the ABCDE-S study, which also assessed single injections of abobotulinumtoxinA 500U to hypertonic upper limb muscles [13]. In both ONTIME and ABCDE-S, significant improvements in MAS for abobotulinumtoxinA vs. placebo were sustained for up to 6 months (Week 24) [13]. The proportion of patients not requiring retreatment by the end of this present study (Week 28, 39.3%) is around five-fold greater than observed in a recent study of adults receiving repeated abobotulinumtoxinA injection for upper limb spasticity at least 6 months post-stroke or post-traumatic brain injury (7.9% at Week 24 or later, after first open-label injection) [20,21]. This suggests a prolonged duration of effect for early intervention with abobotulinumtoxinA. Among cohort differences (e.g., chronic spasticity in a Caucasian population, repeated and >500U abobotulinumtoxinA, and trial design), it should be noted that retreatment in the Gracies et al. (2017) study was by clinicians’ judgement [21], while ONTIME employed a composite index based on patient-centered functional assessments in addition to hypertonicity to evaluate need for re-injection.

While the ONTIME study suggests early abobotulinumtoxinA injection delays symptomatic spasticity development, the effects may not be restorative to maladaptive changes in the brain, due to the finite duration of treatment effect. However, early treatment may modify disease progression before secondary local biomechanical changes occur [22,23,24]. Disease modification at the level of cortical reorganization was demonstrated through functional magnetic resonance imaging in BoNT-A therapy for chronic PSS [25], suggesting preventive potential—and a possible paradigm shift towards early intervention. As spasticity is a form of maladaptive plasticity that progresses over time [13,26], early therapeutic intervention may provide an opportunity to prevent or reduce neurological changes leading to disabling spasticity [23]. Furthermore, early intervention with BoNT-A, combined with adjunctive therapies [27,28], could maximize the impact of treatment on function and tone reduction [18,29]. Although this is a novel approach in spasticity treatment, early intervention using disease-modifying therapies is recognized in current clinical practice guidelines for multiple sclerosis [30], another disease in which a BoNT-A treatment algorithm has been developed for associated spasticity [31].

Early intervention with abobotulinumtoxinA and longer time to symptomatic spasticity progression may positively impact patients’ lives with fewer injections and healthcare provider visits. In addition to patient benefit (demonstrated here by positive impact on global assessment of changes), early abobotulinumtoxinA has shown reduced caregiver burden in previous studies [32,33]. Extending the symptom-free period could have pharmacoeconomic implications, reducing dependency on caregivers and healthcare systems. Cost-effectiveness of BoNT-A in early PSS is a secondary endpoint in an ongoing study [34].

As ONTIME was an exploratory study, the sample size was chosen on the basis of determining the sample size estimation for further confirmatory studies, thus the generalization of efficacy and safety results should be approached with caution. ONTIME study limitations include low numbers of asymptomatic patients at baseline. Additionally, although a trend for improving motor recovery with abobotulinumtoxinA was observed until Week 12, a robust effect was not established due to limited placebo group data. If Fugl–Meyer assessments were performed in all patients until Week 12, or the trial extended, an improvement in active motor function may have occurred. Difficulties in demonstrating motor function improvements with BoNT-A vs. placebo have been experienced previously [18,26]. Future studies should have a longer duration (>24 weeks post-intervention) to observe long-term effects of early abobotulinumtoxinA [29].

## 4. Conclusions

The ONTIME exploratory study demonstrated that early administration of abobotulinumtoxinA, 2–12 weeks post-stroke in patients with spastic paresis, significantly increased time to re-injection criteria fulfilment, compared with placebo, due to prolonged MAS improvements and delayed appearance of spasticity symptoms. Due to the trial design, it was not possible to demonstrate significant differences in motor function. Safety results corresponded to the known profile of abobotulinumtoxinA, and no new safety signals were identified. These results demonstrate that early abobotulinumtoxinA has a good safety profile, may modify the disease course, delay symptom presentation, and lead to healthcare savings. These data provide a basis for future trials to select sample sizes that can confirm these results.

## 5. Materials and Methods

Full details of the ONTIME study (NCT02321436) protocol have been published [35]. ONTIME was conducted in accordance with the Declaration of Helsinki, International Conference on Harmonisation Good Clinical Practice Guidelines, and local regulatory requirements, with approval from relevant independent ethics committee/institutional review boards. Written informed consent was obtained from patients prior to study entry.

### 5.1. Primary Research Question

This study asked whether early abobotulinumtoxinA treatment, in patients with post-stroke ULS, delays time between injection and fulfilment of re-injection criteria (Class I evidence).

### 5.2. Study Design and Participants

ONTIME was a Phase IV, prospective, exploratory, double-blind, randomized, placebo-controlled trial, conducted at four centers in Malaysia, Thailand, Singapore, and the Philippines, initiated in December 2014 and completed in March 2016.

Inclusion criteria included age 18–80 years, 2–12 weeks after first ischemic/hemorrhagic stroke onset, presence of ULS defined as MAS ≥ 2 [36] in PTMG. The investigator selected PTMG at first visit, in agreement with the patient/caregiver. Patients were classified into asymptomatic (increased muscle tone, MAS ≥ 2; Criterion A) and symptomatic cohorts. Symptomatic spasticity (Table S1) was defined as the presence of ≥1 of the following items (Criterion B), in addition to increased muscle tone:

- Impaired passive function (score ≥ 1 on a 4-point Likert scale: 0 = no impact, 1 = mild impact, 2 = moderate impact, 3 = severe impact).
  ○ ‘In general, how much does spasticity impact the following activities of daily living and/or your rehabilitation program: hygiene (i.e., hand, nails, armpit, elbows), dressing the affected limb, positioning the affected limb, splint application or removal?’
- Impaired active function (score ≥ 1 on a 4-point Likert scale, as above).
  ○ ‘In general, how much does spasticity impact the following activities of daily living and/or your rehabilitation program: reaching, grasping, releasing, gripping, holding, bimanual function, manipulating objects, dexterity, fine motor skills, lifting and carrying?’ [19]
- Presence of involuntary movements, which occur during standing up, walking, and transfers (if unable to stand up/walk) (score ≥ 1 on a 4-point Likert scale: 0 = no involuntary movements, 1 = involuntary movements with mild impact on posture and ambulation, 2 = involuntary movements with moderate impact on posture and ambulation, 3 = involuntary movements with severe impact on posture and ambulation).
- Pain (score ≥ 4 on the NPRS: 0 = no pain to 10 = severe disabling pain, with impacts on movement; score of 4 indicates moderate pain) [37].
- Question to each patient was oriented to obtain a relevant answer. Answers were spontaneous and not condition-dependent (e.g., active/passive movements or during night/day). A support could be used to help patients assess pain.
  ○ Average pain intensity over 1 week was collected.

Further details of inclusion and exclusion criteria are detailed in the published protocol [35].

### 5.3. Recruitment and Randomization

Patients were randomized, using an interactive web response system (IWRS) service, to abobotulinumtoxinA or placebo in a 2:1 ratio (maximizing patient numbers receiving active treatment, with sufficient placebo patients to power statistical analyses). The double-blind status of treatment allocation was ensured via separate lists for randomization and treatment numbering. Products were similar in size, color, smell, taste, and appearance. In exceptional instances of an AE, the blind could be broken on an individual basis following review with the Central Department of Pharmacovigilance at Ipsen, or if necessary, by the investigator obtaining a patients’ treatment identification from the IWRS.

### 5.4. Interventions

Patients received intramuscular injections, administered using a 25-gauge needle, of abobotulinumtoxinA 500U or equal volume placebo into selected muscles. AbobotulinumtoxinA and placebo were provided as white lyophilized powders for reconstitution (Dysport^®^, Ipsen Pharma SAS, Paris, France), packed in vials containing 500U BoNT-A hemagglutinin complex or excipients of the investigational product, respectively. Vials were reconstituted with 2.5 mL of preservative-free sodium chloride for injection (0.9%; 200 mL). Doses were administered per muscle according to investigators’ judgements. Recommended dosing regimens were previously published [35]. Most patients participated in occupational and physiotherapy practices.

### 5.5. Efficacy Assessments

#### 5.5.1. Primary Efficacy Assessment

The primary efficacy outcome measure was the time between initial injection (baseline) and visit at which both re-injection criteria were fulfilled (Criterion A: increased muscle tone in PTMG [MAS ≥ 2]; and Criterion B: presence of ≥1 items of symptomatic spasticity).

Re-injection criteria, evaluated at every visit from Week 4, assessed appearance/reappearance of symptomatic spasticity. ‘Appearance’ was assessed for patients asymptomatic and ‘reappearance’ for patients symptomatic at baseline.

#### 5.5.2. Secondary Efficacy Assessments

Secondary assessments included change in muscle tone (MAS) in PTMG, assessed at baseline and each subsequent visit. Active motor function in affected UL was evaluated using Fugl–Meyer assessment (total score, 0–66) for: upper extremity (scored 0–36), wrist (0–10), hand (0–14), and in terms of coordination/speed (0–6) [35,38]. Global assessment of change was investigator-evaluated (5-point Likert scale, ranging from ‘much better’ to ‘much worse’). Fugl–Meyer and global assessment of change were assessed at baseline and each subsequent visit up to, but not including, visit when re-injection criteria were met. Non-drug therapy for ULS was recorded at baseline and each subsequent visit.

### 5.6. Safety Assessments

AEs were monitored and recorded from provision of informed consent until end of participation. TEAEs were reported, events classified as mild, moderate, or severe, and assessed for any causal relationship. Physical examinations and measurement of vital signs were performed by physicians at baseline and all subsequent visits.

### 5.7. Study Schedule

The study schedule has been published previously [35]. The first visit recorded patient demographics, type of stroke, medical and surgical history, and severity of stroke-induced disability (modified Rankin Scale). Visits were scheduled every 2 weeks (±3 days) from Week 4–12, then every 4 weeks (±1 week) to Week 28. Visits up to Week 12 were mandatory, regardless of whether re-injection criteria were met. For patients not fulfilling re-injection criteria by Week 12, subsequent visits were required until re-injection criteria were met or study end.

### 5.8. Statistical Analysis

The sample size (*N* = 42) was chosen for exploratory purposes and not intended for definitive conclusions about efficacy. Statistical analyses were performed using SAS v9.2. Efficacy analyses were performed on the ITT population. Primary endpoint was analyzed by Kaplan–Meier (KM) survival analysis. Median survival time and 95% CIs were estimated for each treatment group using KM product-limit estimation. Data for patients not meeting re-injection criteria at Week 28 were censored at the patient’s last study visit. Treatment differences between abobotulinumtoxinA and placebo groups were tested using two-sided, stratified log-rank and Wilcoxon tests (α = 5%; spasticity status as stratification factor). *p*-values are for exploratory purpose only.

Treatment effect for muscle tone change was assessed using analysis of variance (ANOVA; treatment and symptomatic spasticity status as fixed effects); data are shown as adjusted means, 95% CI, and corresponding *p*-values. Treatment differences in active motor function between abobotulinumtoxinA and placebo groups were assessed using a mixed ANCOVA model on change from baseline (treatment group and symptomatic spasticity status as fixed effects; score value at baseline as covariate). For global assessment of change, differences between treatment groups at last visit were assessed using the Cochran–Mantel–Haenszel statistic, controlling for symptomatic spasticity status. Homogeneity of treatment effect across strata (symptomatic/asymptomatic) was assessed by the Breslow–Day test. Safety analyses were based on safety population and AEs coded using Medical Dictionary for Regulatory Activities (MedDRA) v18.0.

### 5.9. Changes in Conduct of the Study and Planned Analyses

In January 2015, the protocol was amended to correct national legal minimum age in Thailand and clarify data collection methods. A minor statistical update to secondary MAS endpoint analysis was made to replace use of covariance analysis by ANOVA.

## Figures and Tables

**Table 1:** Global assessment of change at last visit (ITT population).

Results, *n* (%)	AbobotulinumtoxinA 500 U (*N* = 28)			Placebo (*N* = 14)		
	Symptomatic Spasticity (*n* = 16)	Asymptomatic Spasticity (*n* = 6)	All (*n* = 22)	Symptomatic Spasticity (*n* = 4)	Asymptomatic Spasticity (*n* = 2)	All (*n* = 6)
Much better	1 (6.3)	1 (16.7)	2 (9.1)	0	0	0
Better	14 (87.5)	4 (66.7)	18 (81.8)	3 (75.0)	2 (100.0)	5 (83.3)
No change	0	1 (16.7)	1 (4.5)	1 (25.0)	0	1 (16.7)
Worse	1 (6.3)	0	1 (4.5)	0	0	0
Much worse	0	0	0	0	0	0
Cochran-Mantel-Haenszel *p*-value = 0.6128						

No data are available for 14 patients as they met re-injection criteria at Week 4. Cochran–Mantel–Haenszel *p*-value represents the strength of the association between treatment and global assessments of changes at the last visit, adjusted for symptomatic status at baseline. ITT, intention-to-treat.

**Table 2:** Summary of adverse events (safety population).

	**AbobotulinumtoxinA 500 U (*N* = 28)**	**Placebo (*N* = 14)**	**All patients (*N* = 42)**
Any adverse events	8 (28.6), *17*	4 (28.6), *6*	12 (28.6), *23*
Any serious adverse events	3 (10.7), *4*	0	3 (7.1), *4*
Any TEAEs	7 (25.0), 16	4 (28.6), *6*	11 (26.2), *22*
Intensity of TEAEs			
Severe	1 (3.6), 2	0	1 (2.4), *2*
Moderate	6 (21.4), *11*	1 (7.1), *1*	7 (16.7), *12*
Mild	3 (10.7), *3*	3 (21.4), *5*	6 (14.3), *8*
Related TEAEs	0	0	0
**Reported TEAEs:**			
Head injury	2 (7.1), *2*	0	2 (4.8), *2*
Insomnia	2 (7.1), *2*	0	2 (4.8), *2*
Fall	1 (3.6), *1*	1 (7.1), *1*	2 (4.8), *2*
Urinary tract infection	1 (3.6), *1*	1 (7.1), *2*	2 (4.8), *3*
Asthma	1 (3.6), *1*	0	1 (2.4), *1*
Constipation	1 (3.6), *1*	0	1 (2.4), *1*
Cough	1 (3.6), *1*	0	1 (2.4), *1*
Hypertensive crisis	1 (3.6), *1*	0	1 (2.4), *1*
Hypokalemia	1 (3.6), *1*	0	1 (2.4), *1*
Pain	1 (3.6), *1*	0	1 (2.4), *1*
Pneumonia	1 (3.6), *1*	0	1 (2.4), *1*
Pyrexia	1 (3.6), *1*	0	1 (2.4), *1*
Tachycardia	1 (3.6), *1*	0	1 (2.4), *1*
Vomiting	1 (3.6), *1*	0	1 (2.4), *1*
Dizziness	0	1 (7.1), *1*	1 (2.4), *1*
Epistaxis	0	1 (7.1), *1*	1 (2.4), *1*
Neuralgia	0	1 (7.1), *1*	1 (2.4), *1*

All values are presented as the number of patients (percentage of patients), *number of occurrences*. Medical Dictionary for Regulatory Activities (MedDRA) Version 18.0. TEAE, treatment-emergent adverse event.

## Supplementary Materials

The following are available online at http://www.mdpi.com/2072-6651/10/7/253/s1, Table S1: Baseline characteristics for patients in the ONTIME study (ITT population), Table S2: Concomitant non-drug therapy use (safety population), Table S3: Concomitant post-stroke medications by therapeutic class (safety population).
