# Supplementary material for: Early AbobotulinumtoxinA (Dysport®) in Post-Stroke Adult Upper Limb Spasticity: ONTIME Pilot Study
Source: Toxins (Basel). 2018 Jun 21;10(7):253. doi: 10.3390/toxins10070253 (PMC6070912; doi:10.3390/toxins10070253)
Supplement: Supplementary file 1 [file toxins-10-00253-s001.pdf]

Supplementary Materials: Early AbobotulinumtoxinA (Dysport®) in Post-Stroke Adult Upper Limb Spasticity: ONTIME Pilot Study

Raymond L Rosales, Jovita Balcaitiene, Hugues Berard, Pascal Maisonobe, Khean Jin Goh, Witsanu Kumthornthip, Mazlina Mazlan, Lydia Abdul Latif, Mary Mildred D. Delos Santos, Chayaporn Chotiyarnwong, Phakamas Tanvijit, Odessa Nuez and Keng He Kong

Table S1. Baseline characteristics for patients in the ONTIME study (ITT population).

	**AbobotulinumtoxinA 500 U (*N* = 28)**	**Placebo (*N* = 14)**	**All patients (*N* = 42)**
Age in years, mean (SD) [range]	61.5 (13.2) [32, 80]	56.5 (9.7) [33, 72]	59.8 (12.3) [32, 80]
Sex, *n* (%)			
Male	23 (82.1)	10 (71.4)	33 (78.6)
Female	5 (17.9)	4 (28.6)	9 (21.4)
Country, *n* (%)			
Malaysia	3 (10.7)	1 (7.1)	4 (9.5)
Philippines	11 (39.3)	4 (28.6)	15 (35.7)
Singapore	9 (32.1)	5 (35.7)	14 (33.3)
Thailand	5 (17.9)	4 (28.6)	9 (21.4)
Time in weeks since stroke diagnosis, mean (SD) [range]	6.18 (2.87) [2.3, 11.7]	6.52 (2.53) [2.9, 11.3]	6.29 (2.73) [2.3, 11.7]
Type of stroke, *n* (%)			
Ischemic	20 (71.4)	10 (71.4)	30 (71.4)
Hemorrhagic	8 (28.6)	4 (28.6)	12 (28.6)
Arm affected, *n* (%)			
Left	16 (57.1)	8 (57.1)	24 (57.1)
Right	12 (42.9)	6 (42.9)	18 (42.9)
Both	0	0	0
mRS score at baseline, mean (SD) [range]	3.9 (0.5) [3, 5]	3.8 (0.4) [3, 4]	3.9 (0.5) [3, 5]
Disposition of patients by mRS score at baseline, *n* (%)			
0-No symptoms	0	0	0
1-No significant disability	0	0	0
2-Slight disability	0	0	0
3-Moderate disability	5 (17.9)	3 (21.4)	8 (19.0)
4-Moderately severe disability	20 (71.4)	11 (78.6)	31 (73.8)
5-Severe disability	3 (10.7)	0	3 (7.1)
Primary targeted muscles, *n* (%)			
Elbow flexors	20 (71.4)	11 (78.6)	31 (73.8)
Elbow pronators	4 (14.3)	0	4 (9.5)
Wrist flexors	4 (14.3)	2 (14.3)	6 (14.3)
Finger flexors	0	1 (7.1)	1 (2.4)
Spasticity at baseline, *n* (%)			
Symptomatic	22 (78.6)	10 (71.4)	32 (76.2)
Asymptomatic	6 (21.4)	4 (28.6)	10 (23.8)

MAS, mean (SD) [range]	2.11 (0.31) [2.0, 3.0]	2.14 (0.36) [2.0, 3.0]	2.12 (0.33) [2.0, 3.0]
Passive function (Likert scale), *n* (%)			
0-No impact	8 (28.6)	7 (50.0)	15 (35.7)
1-Mild impact	6 (21.4)	4 (28.6)	10 (23.8)
2-Moderate impact	11 (39.3)	1 (7.1)	12 (28.6)
3-Severe impact	3 (10.7)	2 (14.3)	5 (11.9)
Active function (Likert scale), *n* (%)			
0-No impact	11 (39.3)	7 (50.0)	18 (42.9)
1-Mild impact	3 (10.7)	1 (7.1)	4 (9.5)
2-Moderate impact	10 (35.7)	3 (21.4)	13 (31.0)
3-Severe impact	4 (14.3)	3 (21.4)	7 (16.7)
Involuntary movements (Likert scale), *n* (%)			
0-No involuntary movements	15 (53.6)	7 (50.0)	22 (52.4)
1-Mild impact	6 (21.4)	3 (21.4)	9 (21.4)
2-Moderate impact	6 (21.4)	3 (21.4)	9 (21.4)
3-Severe impact	1 (3.6)	1 (7.1)	2 (4.8)
Numeric Pain Rating Scale			
Score of > 4, *n* (%)<br>Mean score (SD) [range]	12 (42.9)<br>3.1 (3.2) [0, 10.0]	4 (28.6)<br>3.1 (2.7) [0, 10.0]	16 (38.1)<br>3.1 (3.0) [0, 10.0]

ITT, intention-to-treat; MAS, Modified Ashworth Scale; mRS, modified Rankin Scale; SD, standard deviation.

Table S2. Concomitant non-drug therapy use (safety population).

	**AbobotulinumtoxinA 500 U (*N* = 28)**	**Placebo (*N* = 14)**	**All patients (*N* = 42)**
Any concomitant non-drug therapies, *n* (%)	25 (89.3)	14 (100.0)	39 (92.9)
Occupational therapy	8 (28.6)	5 (35.7)	13 (31.0)
Physiotherapy	22 (78.6)	14 (100.0)	36 (85.7)
Duration in days of physiotherapy, mean (SD) [range]	157.9 (59.2) [70, 230]	126.1 (55.0) [59, 271]	-

SD, standard deviation.

Table S3. Concomitant post-stroke medications by therapeutic class (safety population).

**Therapeutic class, *n* (%)**	**AbobotulinumtoxinA 500 U (*N* = 28)**	**Placebo (*N* = 14)**	**All patients (*N* = 42)**
Any concomitant medication	3 (10.7)	3 (21.4)	6 (14.3)
Muscle relaxants	1 (3.6)	2 (14.3)	3 (7.1)
Antiepileptics	1 (3.6)	1 (7.1)	2 (4.8)
Analgesics	1 (3.6)	0	1 (2.4)
Lipid-modifying agents	1 (3.6)	0	1 (2.4)

Prior and concomitant medications were recorded at the each study visit.
